# Supplementary figures and images for: Aquaporin splice variation differentially modulates channel function during marine teleost egg hydration
Source: PLoS One. 2023 Nov 27;18(11):e0294814. doi: 10.1371/journal.pone.0294814 (PMC10681232; doi:10.1371/journal.pone.0294814)

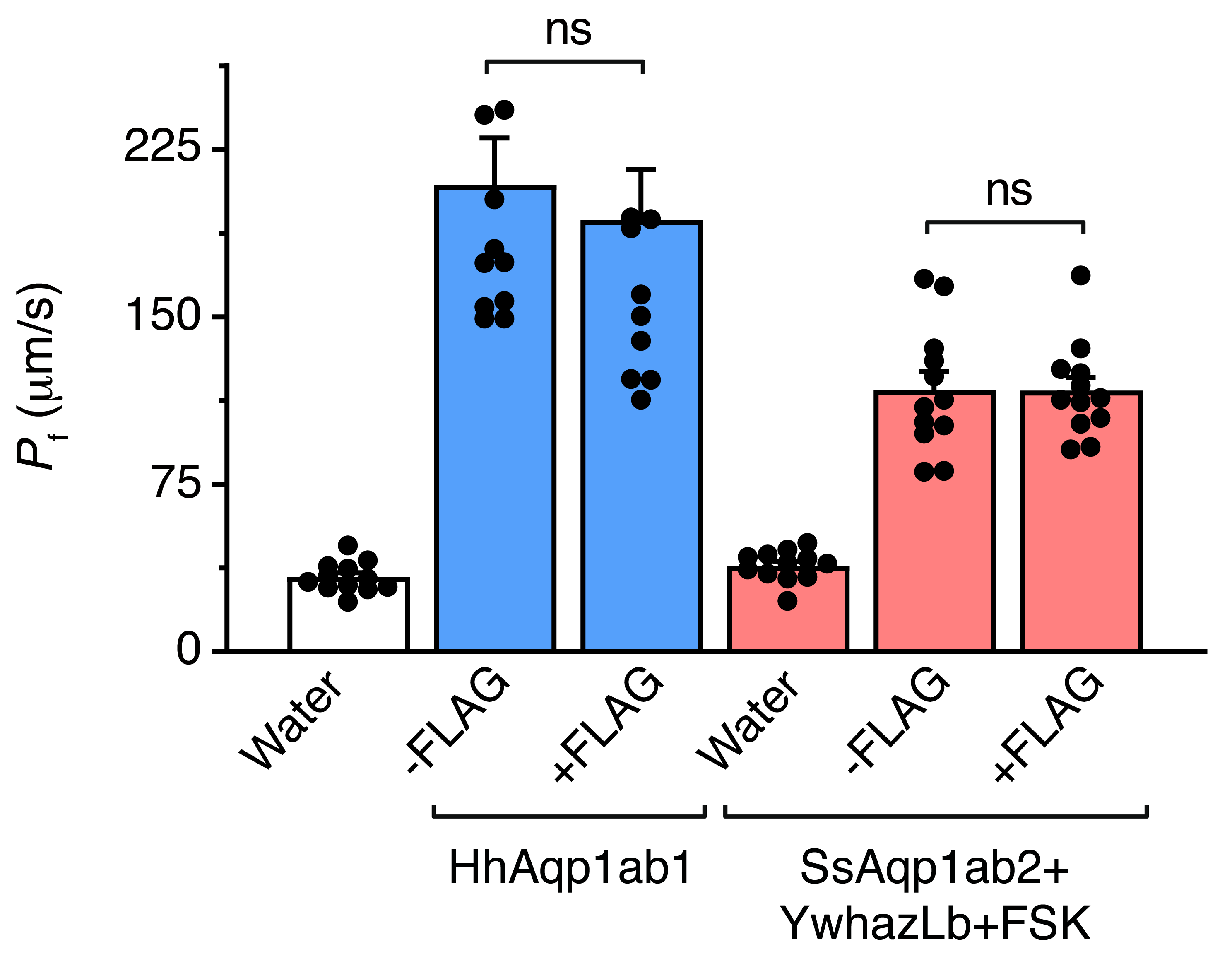

Supplement: S1 Fig — Pf of X. laevis oocytes injected with water (W, controls), or expressing non-tagged or Flag-tagged Atlantic halibut HhAqp1ab1-WT or sole SsAqp1ab2-WT. Oocytes injected with SsAqp1ab2-WT were co-injected with non-tagged halibut YwhazLb and exposed to FSK. The data are the mean ± SEM (n = 12 oocytes per treatment, indicated with dots above each bar). (TIF) [file pone.0294814.s001.tif]

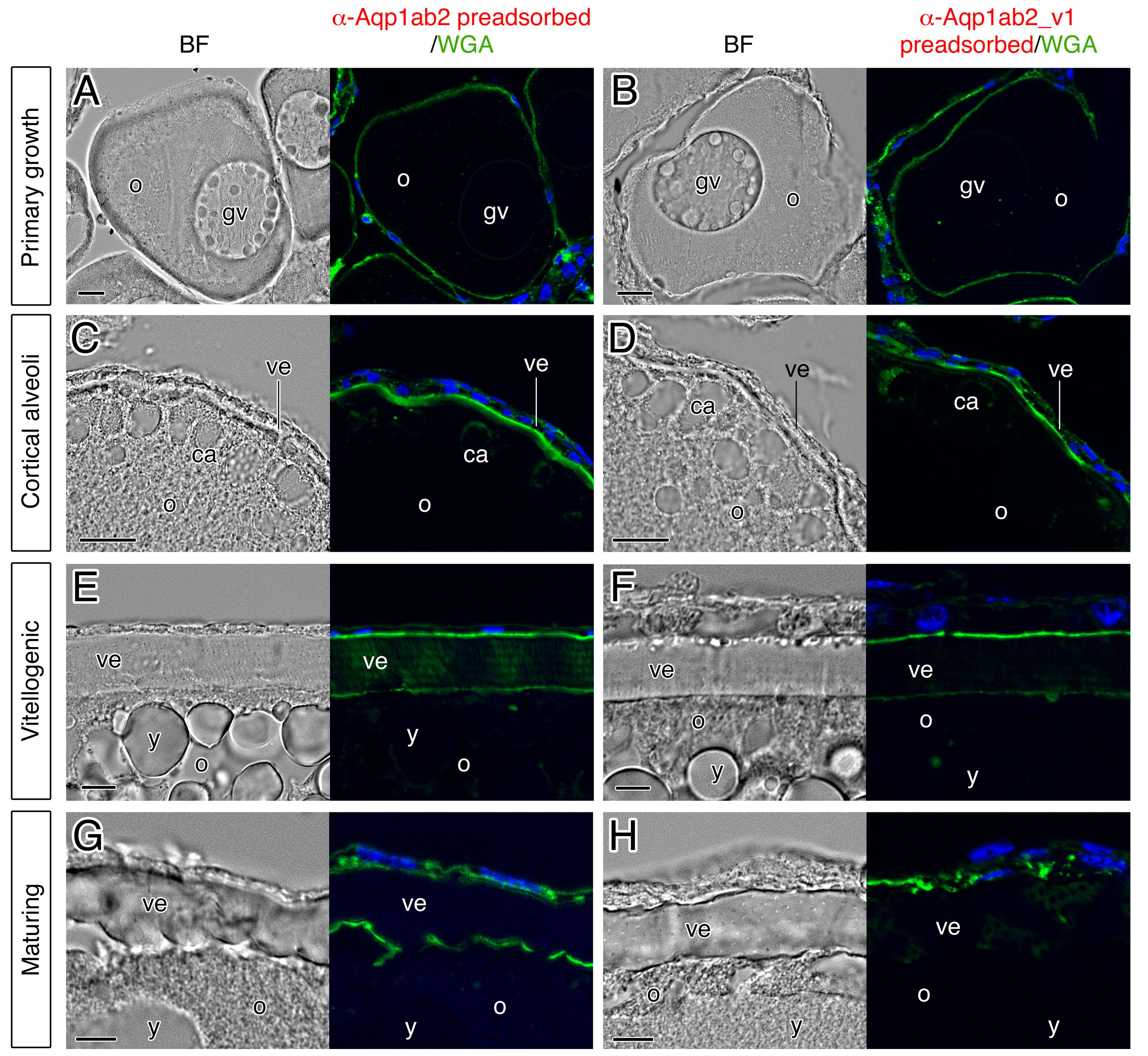

Supplement: S2 Fig — Histological sections were incubated with Aqp1ab2-Nt and Aqp1ab2_v1 antisera preabsorbed with the immunizing peptides, indicating the specificity of the antibodies. Abbreviations: o, oocyte; y, yolk globule; gv, germinal vesicle; ve, vitelline envelope; cp, capillary; ca, cortical alveoli. Scale bars, 10 μm. (TIF) [file pone.0294814.s002.tif]

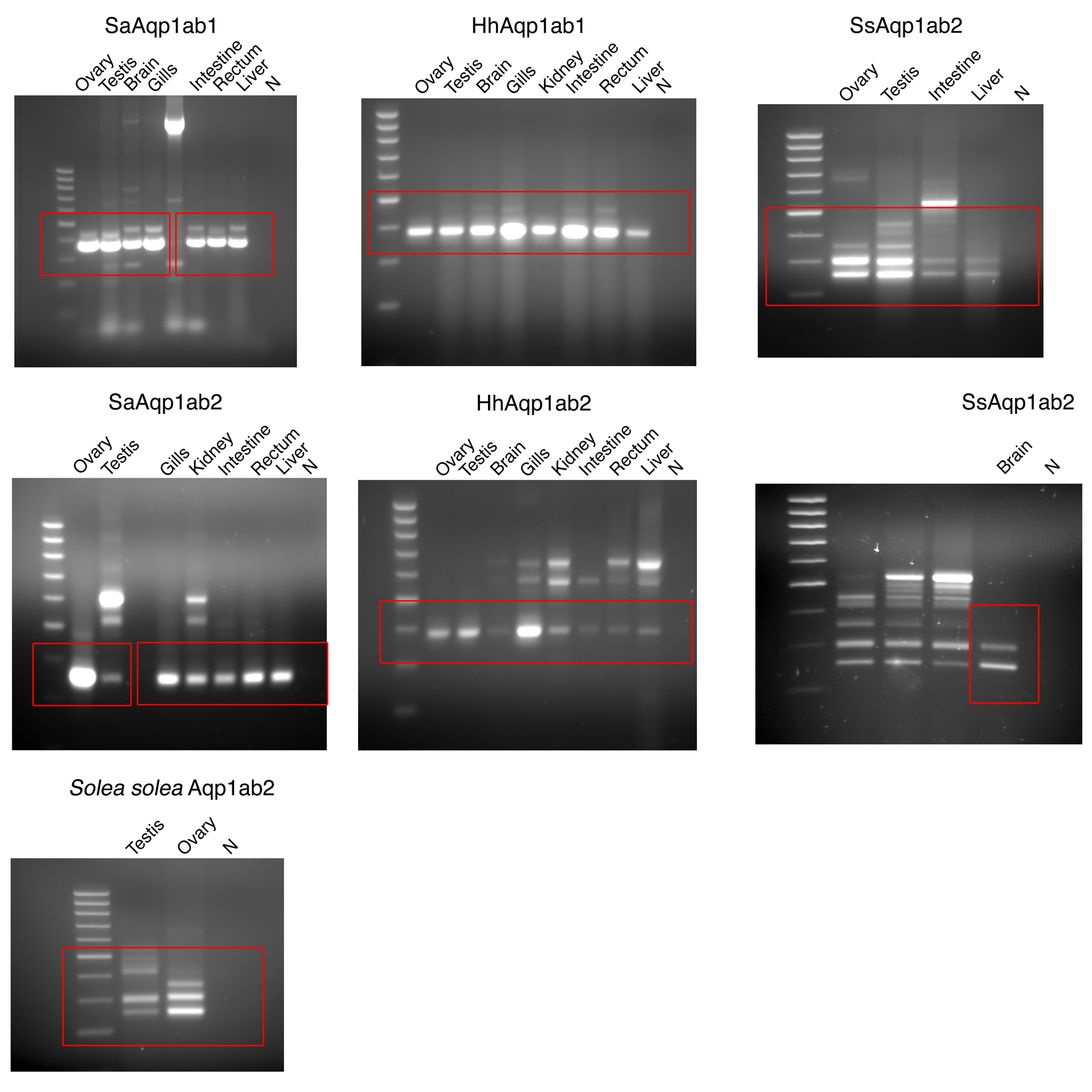

Supplement: S1 File — (TIF) [file pone.0294814.s003.tif]

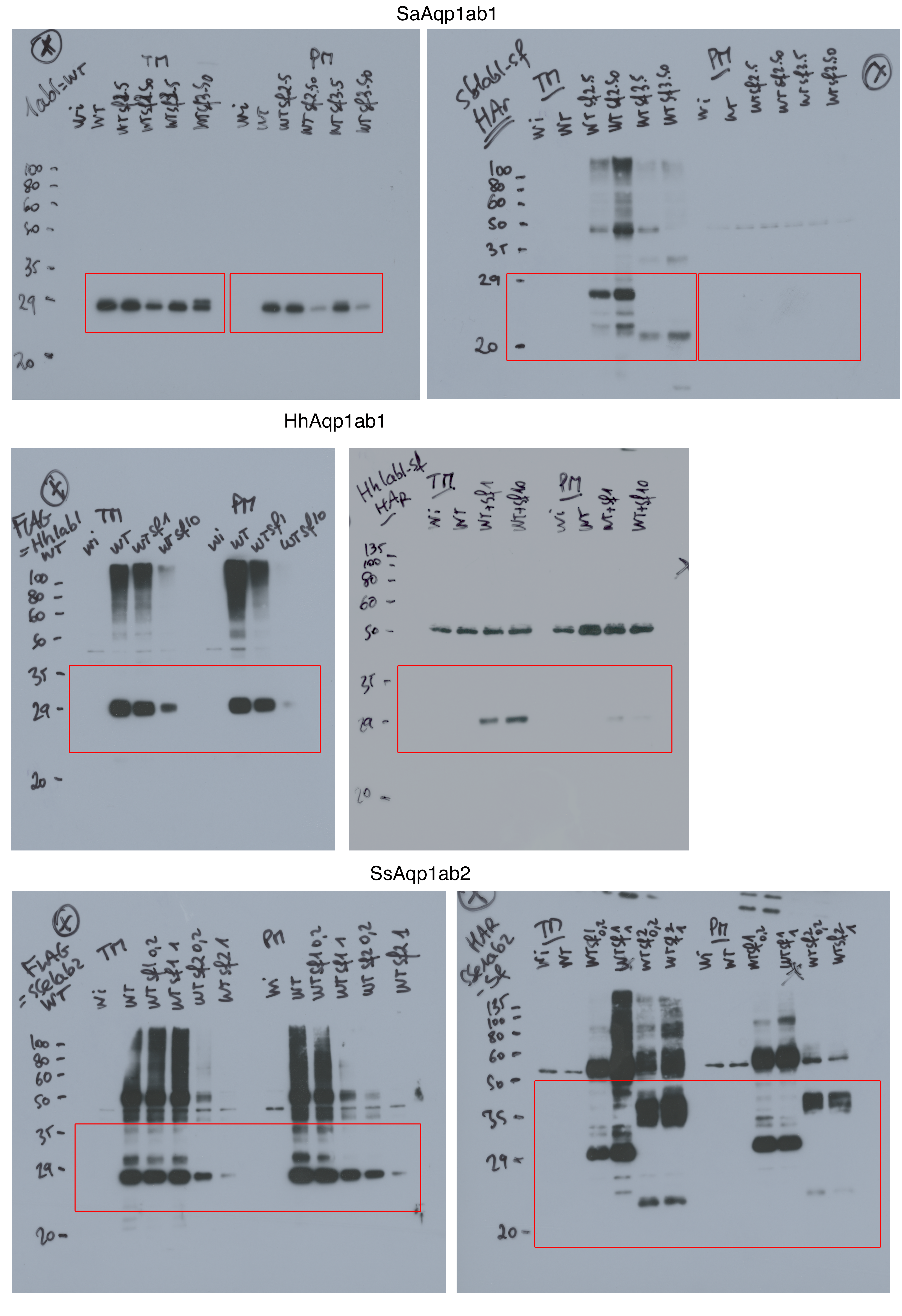

Supplement: S2 File — (TIF) [file pone.0294814.s004.tif]

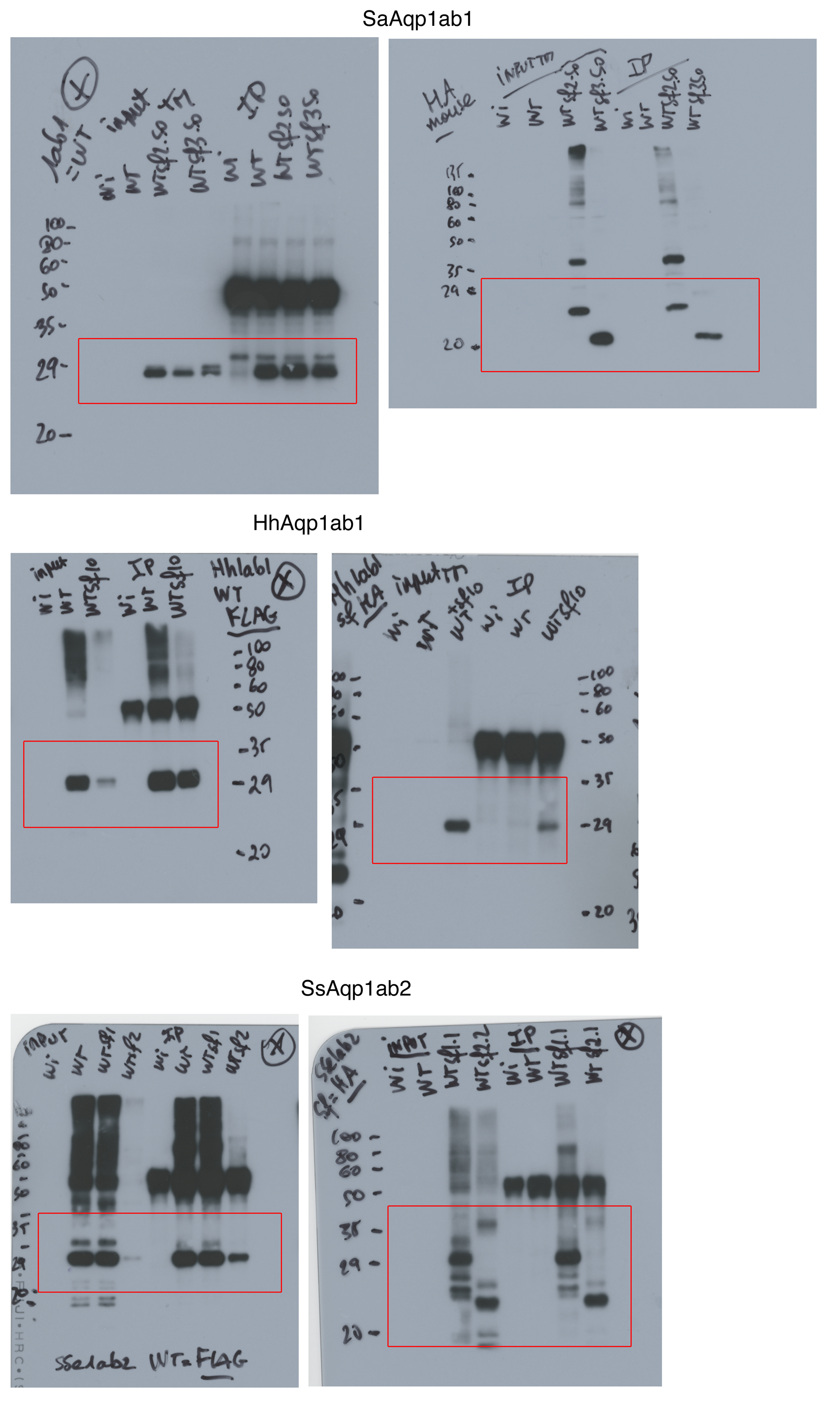

Supplement: S3 File — (TIF) [file pone.0294814.s005.tif]

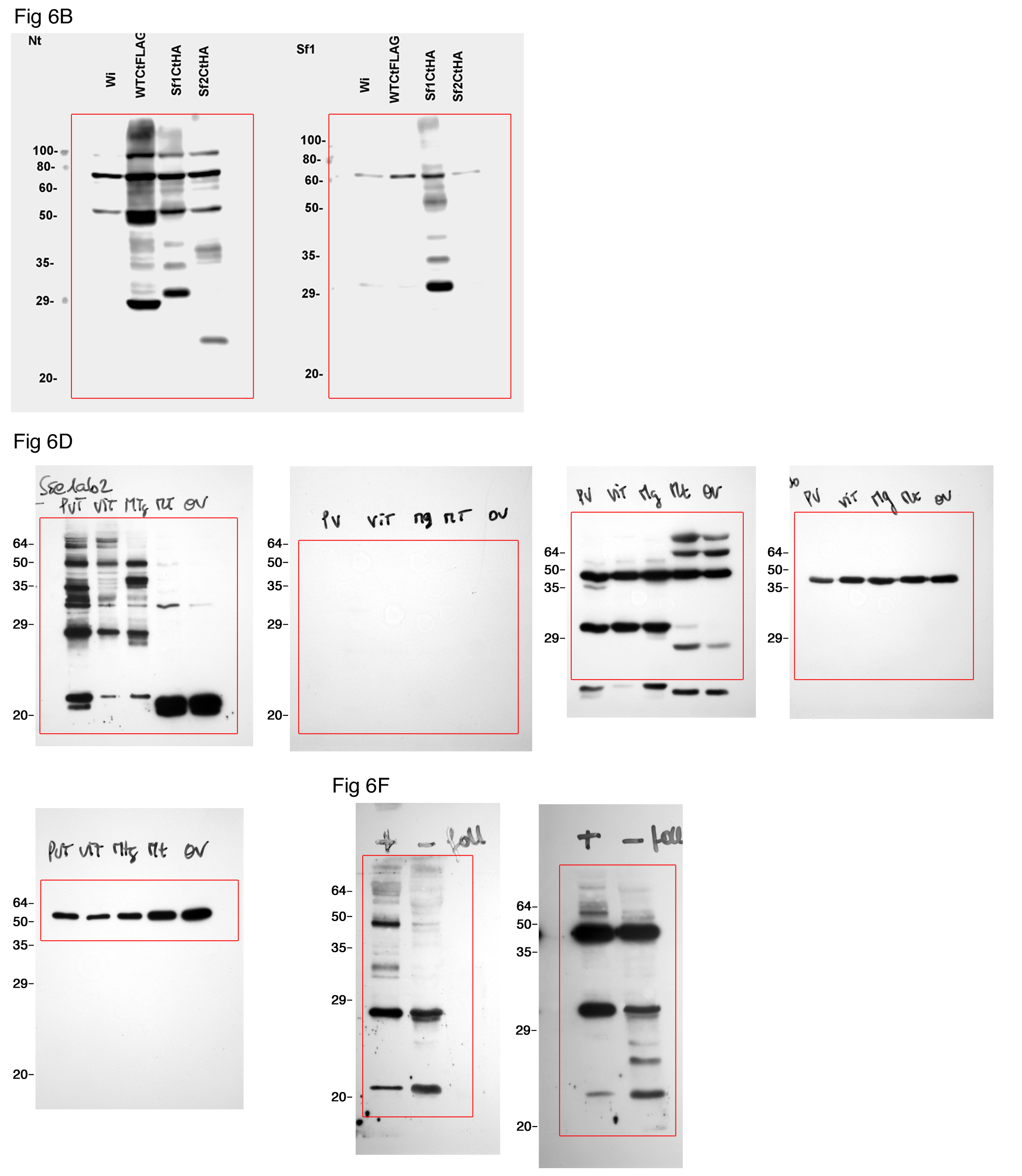

Supplement: S4 File — (TIF) [file pone.0294814.s006.tif]

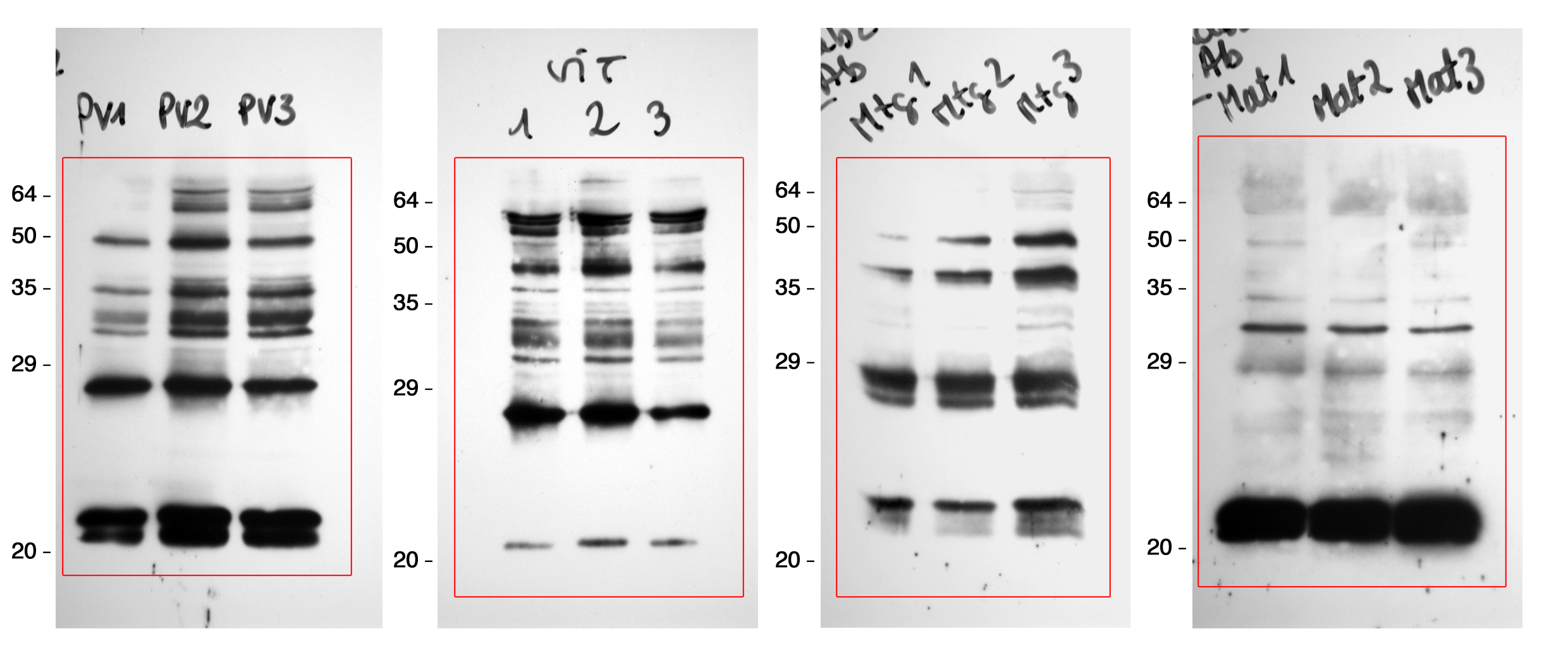

Supplement: S5 File — (TIF) [file pone.0294814.s007.tif]
