# Supplementary material for: Aquaporin splice variation differentially modulates channel function during marine teleost egg hydration
Source: PLoS One. 2023 Nov 27;18(11):e0294814. doi: 10.1371/journal.pone.0294814 (PMC10681232; doi:10.1371/journal.pone.0294814)
Supplement: S1 Table — (DOC) [file pone.0294814.s009.doc]

**Oligonucleotide primers employed for the RT-PCR screening of splice forms expression of *aqp1ab*-type genes in teleosts**

| **Species** | **Gene** | **GenBank accession no.** | **Forward (F) / Reverse (R)** | **Amplicon (bp)** |
| --- | --- | --- | --- | --- |
| *Sparus aurata* | *aqp1ab1* | AY626938 | F: CTGGCTATTGGGCTGTCAGT  R: AGCCATATTGAAAGCTTTTCTGC | 375 |
|  | *aqp1ab2* | MW960021 | F: TTTGGCGTAGAATTCCTGCT  R: CGAATGGCCATTTTCCATTGT | 356 |
| *Hippoglossus hippoglossus* | *aqp1ab1* | MW960022 | F: CGAGTCATGTCAAGGGCTTT  R: GCCGACAGCCAGAAAAGTTA | 380 |
|  | *aqp1ab2* | HQ185295 | F: GCCATTGAATTCCTGCTCAC  R: TTCACATCCTCAACGTCGTC | 383 |
| *Solea senegalensis /*  *Solea solea* | *aqp1ab2* | AY626941 | F: CGCAGTCACTGACAAACGAC  R: CTCTGAAGGACTCGTGACTGG | 254 |
